# Terminal Schwann Cell Aging: Implications for Age-Associated Neuromuscular Dysfunction

**DOI:** 10.14336/AD.2020.0708

**Published:** 2021-04-01

**Authors:** Sandra Fuertes-Alvarez, Ander Izeta

**Affiliations:** ^1^Biodonostia, Tissue Engineering Group, Paseo Dr. Begiristain, s/n, San Sebastian 20014, Spain.; ^2^Tecnun-University of Navarra, School of Engineering, Department of Biomedical Engineering and Science, Paseo Mikeletegi, 48, San Sebastian 20009, Spain.

**Keywords:** aging, neuromuscular junction (NMJ), terminal Schwann cell (tSC), muscle denervation, sarcopenia, frailty, peripheral nervous system

## Abstract

Action potential is transmitted to muscle fibers through specialized synaptic interfaces called neuromuscular junctions (NMJs). These structures are capped by terminal Schwann cells (tSCs), which play essential roles during formation and maintenance of the NMJ. tSCs are implicated in the correct communication between nerves and muscles, and in reinnervation upon injury. During aging, loss of muscle mass and strength (sarcopenia and dynapenia) are due, at least in part, to the progressive loss of contacts between muscle fibers and nerves. Despite the important role of tSCs in NMJ function, very little is known on their implication in the NMJ-aging process and in age-associated denervation. This review summarizes the current knowledge about the implication of tSCs in the age-associated degeneration of NMJs. We also speculate on the possible mechanisms underlying the observed phenotypes.

## 1. Introduction

The neuromuscular junction (NMJ) is the synaptic interface through which motor neurons innervate muscle fibers [1, 2]. Of note, not only motor nerves but also sympathetic neurons innervate skeletal muscles, and this is essential to control autonomic functions [3-6]. The correct function of the NMJ is thus essential for muscle contraction, and the impairment of synaptic activity in skeletal muscles gives rise to neuromuscular disorders such as amyotrophic lateral sclerosis (ALS) [7, 8] and myasthenia gravis [9-11]. As it happens in the rest of the organism, NMJ function may also be impaired during normal aging [12-16]. Age-associated NMJ impairment ultimately results in muscle atrophy and declined muscle mass and strength (i.e. sarcopenia and dynapenia), features that have been associated to physical frailty [17-19].

The NMJ presents a characteristic pretzel-like structure [14] which is composed of five essential elements: (i) presynaptic motor nerve terminals; (ii) postsynaptic endplates in muscle fiber membranes (where acetylcholine receptors -AChRs- are anchored); (iii) basal lamina, the extracellular matrix located in the synaptic cleft [20, 21]; (iv) terminal Schwann cells (tSCs), which cover the nerve-muscle junctions (2-5 tSCs per NMJ) [22, 23], and (v) fibroblast-like cells known as kranocytes or perisynaptic fibroblasts, which cap the NMJs from above the tSCs, thus covering the end-plate area in its entirety [24, 25]. Of these NMJ components, kranocytes are currently the least studied. It is believed that they may play essential roles in NMJ maintenance and regeneration, since they seem to proliferate and spread upon nerve injury, before tSC sprouts develop [22, 24-26].

tSCs (also known as perisynaptic Schwann cells) represent the glial component of NMJs, capping the presynaptic nerve terminal aligned with postsynaptic AChRs. The tSCs are a subtype of non-myelinating Schwann cells (nmSCs). During embryonic development, Schwann cell precursors (SCPs) derive from the neural crest. SCPs then develop into immature Schwann cells that persist until birth. Postnatally, immature SCs differentiate into myelinating (m) and non-myelinating (nm) Schwann cells (SCs). mSCs form myelin sheaths that wrap thicker (Ø>1mm) axons, thus permitting an increase in their conduction speed. nmSCs do not produce myelin and are divided into two groups: Remak cells, associated to thinner (Ø<1mm) axons, and tSCs associated to NMJs [27-29]. tSCs are essential in the synaptic function of adult NMJs as well as during formation, maintenance and remodeling of NMJs [30-34]. As it would be expected from their key structural role in the healthy NMJ, tSC dysfunction during pathological conditions may be at the origin of some neuromuscular diseases [7, 35-39].

**Figure 1.**
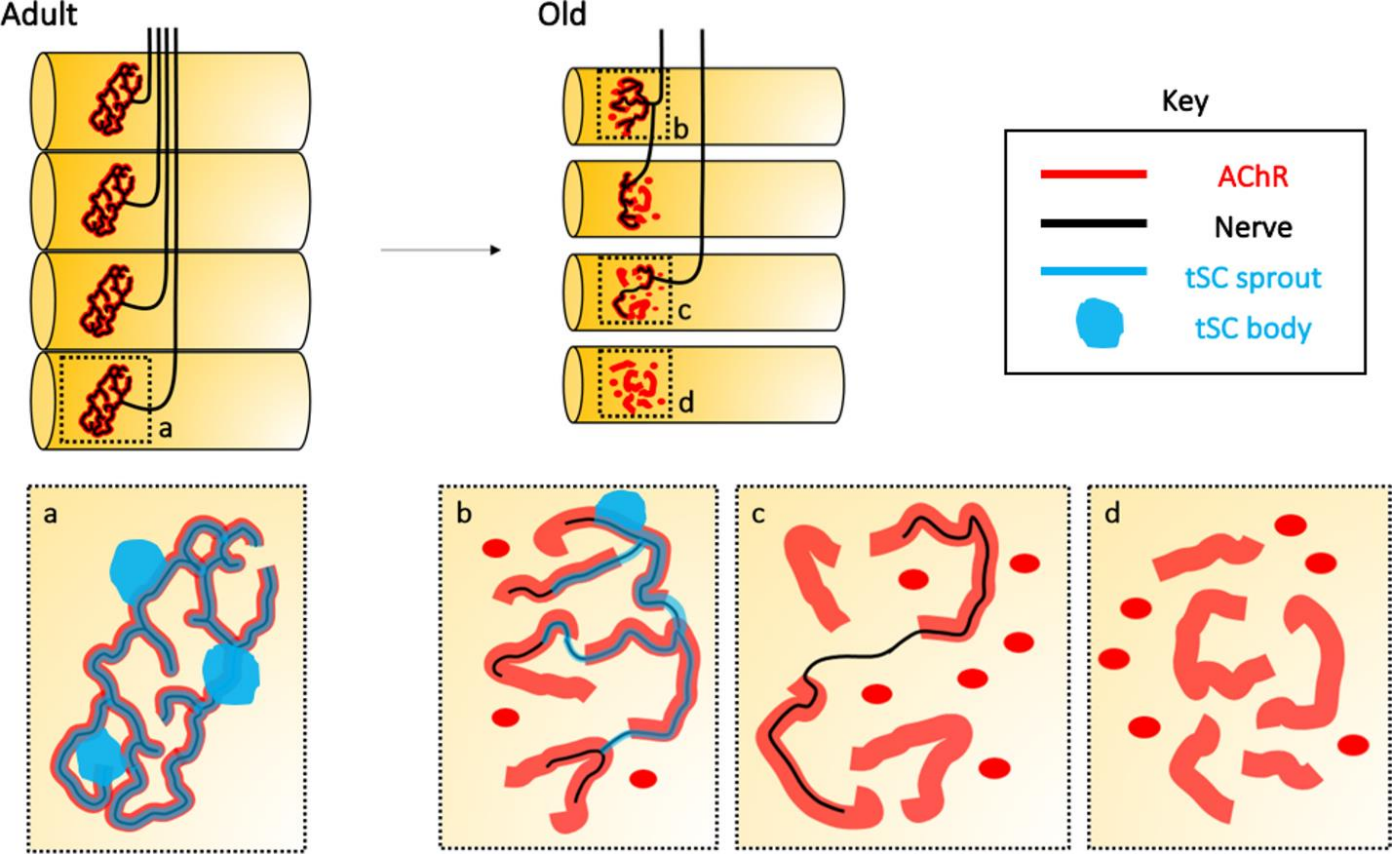
Age-associated changes in the neuromuscular junction (NMJ) structure. In adults, each muscle fiber is innervated by a single motor neuron-associated endplate that constitutes the NMJ. This correlation is lost upon aging, and fibers may become partially or completely denervated, and often reinnervated by bridges arising from neighboring NMJs. (a) Adult NMJs typically show a branched morphology known as “pretzel-like” structure, where acetylcholine receptor (AChR) immunoreactivity (red line) fully colocalizes with axonal branches (black line) covered by terminal Schwann cells (tSCs; blue line and blue dots). (b-d) Upon aging, pretzel-like structures are fragmented into multiple AChR-immunoreactive (red) “islands” (b). Aged NMJs become partially (c; black lines representing nerves) or completely (d) denervated. tSCs may cover aged NMJs only partially, and present aberrant processes (b; blue lines). More often, NMJs simply lose any associated tSCs (c-d).

Similarly, numerous studies have addressed NMJ degeneration during sarcopenia and aging [7, 14, 40-44]. In adults, the NMJs (one per muscle fiber) show characteristic pretzel-like structures, which are fully innervated and covered by tSCs (Fig. 1a). In contrast, aged NMJs present a number of aberrations, such as: (i) endplate fragmentation, i.e. loss of pretzel-like structure and redistribution of the AChR immunoreactivity into smaller “islands” (Fig. 1b-d); (ii) reduced AChR density and partial coverage of the NMJ by tSC (Fig. 1b); and (iii) partial (Fig. 1c) or even complete (Fig. 1d) denervation of the NMJ [14, 45-47].

A fair number of recent articles has reviewed the process of age-related degeneration of NMJs from diverse points of view [14, 48-51]. However, loss of function of aged-tSCs and its implications in the NMJ aging process are less understood. This review is focused on collecting the relatively sparse evidence on age-associated tSC changes during normal aging. By extrapolating from regeneration or pathophysiological paradigms that affect the NMJs, we go on to suggest possible pathways involved in the aging of tSCs, and finally speculate on its potential link with the development of sarcopenia. Thus, this review article intends to give our personal perspective (from a “tSchwanncentric” point of view) of age-related degeneration of NMJs.

## 2.Terminal Schwann cells (tSCs) in the adult NMJ

tSCs are specialized glial cells localized over the NMJs, which regulate not only the establishment and maintenance of NMJs [34] but also the synapse efficacy between motor neuron and muscle fibers and terminal nerve guidance during NMJ-reinnervation [33, 52].

Each mature NMJ presents between 3-5 tSCs, the number correlating with the end plate size [53-55]. As aforementioned, these cells cap the synaptic zone and are essential for the maturation and long-term maintenance of NMJs [33, 56, 57]. Importantly, tSCs are able to sense and modulate synaptic activity [58, 59] to ensure an appropriate signal strength and an efficient transmission [33]. Targeted ablation of tSCs gives rise to nerve terminal retraction, loss of pretzel-like structure, fragmentation of the NMJ and reduced neuromuscular transmission [30, 57, 60-66].

Throughout adult life, the synaptic neuromuscular connections undergo remodeling, which seems to be more prominent in frogs than in mammals [52, 67-69]. However, the dynamic behavior of mammalian NMJs is well known [33, 67, 68, 70]. Both nerves and tSCs may sprout beyond the AChR immunoreactivity limits [67, 71-74], perhaps mimicking the reshaping process observed during nerve regeneration and NMJ-reinnervation. Here, tSCs form sprouts (a.k.a. “bridges” or “escaped fibers”) through which tSCs and axons reach and connect with adjacent denervated endplates [54, 72, 73, 75, 76]. Overall, these results indicate that mammalian mature NMJs become plastic upon nerve injury-mediated denervation. Despite its high relevance for understanding age-associated muscular dysfunction, plasticity of the NMJ during sarcopenia and aging is still poorly understood.

tSCs are able to sense the synaptic communication and modulate synaptic properties [77-79]. Once the action potential arrives to the NMJ, vesicles loaded with acetylcholine (ACh) reach the nerve terminal membrane and release ACh into the synaptic cleft, activating the nicotinic ACh receptors (nAChRs) in the postsynaptic endplate to initiate muscle contraction. ACh molecules in the synaptic cleft are also detected by muscarinic ACh receptors (mAChR) in the tSC membrane [33, 52, 80, 81], allowing tSCs to detect the signal as well. In response to peripheral nerve stimulation, tSCs increase their levels of cytoplasmic Ca^2+^ [58, 59, 78, 80, 82-84]. Differences in synaptic signal are detected through the expression of the A_2A_ receptors and A_1_ receptors, which provide specific feedback: the sustained potentiation is mediated by A_2A_ receptors and the sustained depression is mediated by A_1_ receptors [85]. Moreover, tSCs differentiate synaptic activity in slow-twitch and fast-twitch muscle, and respond appropriately to each type of synapse: the tSC Ca^2+^ response obtained at fast-twitch synapses is larger and displays faster kinetics as compared to the response at slow twitch synapses [79].

Altogether, these studies demonstrate that tSCs sense and respond to the synaptic activity at the NMJ. Furthermore, tSCs are also capable to modulate the properties of the synaptic communication, releasing gliotransmitters (i.e. molecules with potential to modulate neuronal activity) as glutamate, prostaglandins or nitric oxide to the synaptic cleft [82, 86-94]. Hence, tSCs act not only as sensors of the neuromuscular transmission, but also as regulators of the synaptic activity.

This complex crosstalk unveils tSCs as essential elements of the NMJs, sensing and modulating synaptic signals as well as establishing “bridges” to adjacent endplates when necessary [72, 73, 75, 95]. However, changes in the tSC synaptic sensor and modulator capacity under pathological conditions have barely been addressed [8]. Similar unknowns surround tSC behavior in sarcopenia and aging [96].

## 3.Terminal Schwann cells (tSCs) in the aged NMJ

Morphological plasticity of tSCs is widely seen in neuromuscular disease and upon nerve injury (i.e. after nerve cutting, crushing, resecting, etc.) [7, 35, 36, 72, 73, 75, 76, 97-104]. However, little is known about the response of tSCs to an aged-NMJ environment [45, 96, 105-107]. As a consequence, the implication of tSCs in NMJ-degeneration during aging and sarcopenia remains poorly understood.

Age-associated degeneration of NMJs has been documented both in animal models and humans, although if human NMJs degenerate upon normal aging is currently debated (see below) [14, 108-111]. During aging, the NMJ loses its characteristic “pretzel-like” structure, acquiring a more fragmented appearance [14, 45, 110, 112] (Fig. 1). Endplates acquire dotted and apparently unstructured shape, although different phenotypes are observed depending on the muscle studied [16, 45, 112-114]. The level of endplate disorganization seems to correlate with the accumulation of degeneration and regeneration cycles in muscle fibers. In healthy individuals, damaged muscle fibers are quickly replaced and reinnervated. However, the regenerated endplates show structural alterations [46]. For instance, regenerated endplates occupy larger areas than the original structure and thus present decreased density of AChR expression [47, 113]. Moreover, during aging the number of postsynaptic folds are reduced [111], once again pointing to impairment of signaling and synaptic activity in the old individuals. Depending on the muscle analyzed, different frequencies of denervated NMJs may be observed in old individuals, both in mice [45, 112] and humans [115]. Generally, denervation compromises muscle function [116-118]. However, a decrease in muscle functionality is not seen in all muscles [16]. This variability among muscle groups remains to be explained.

**Figure 2.**
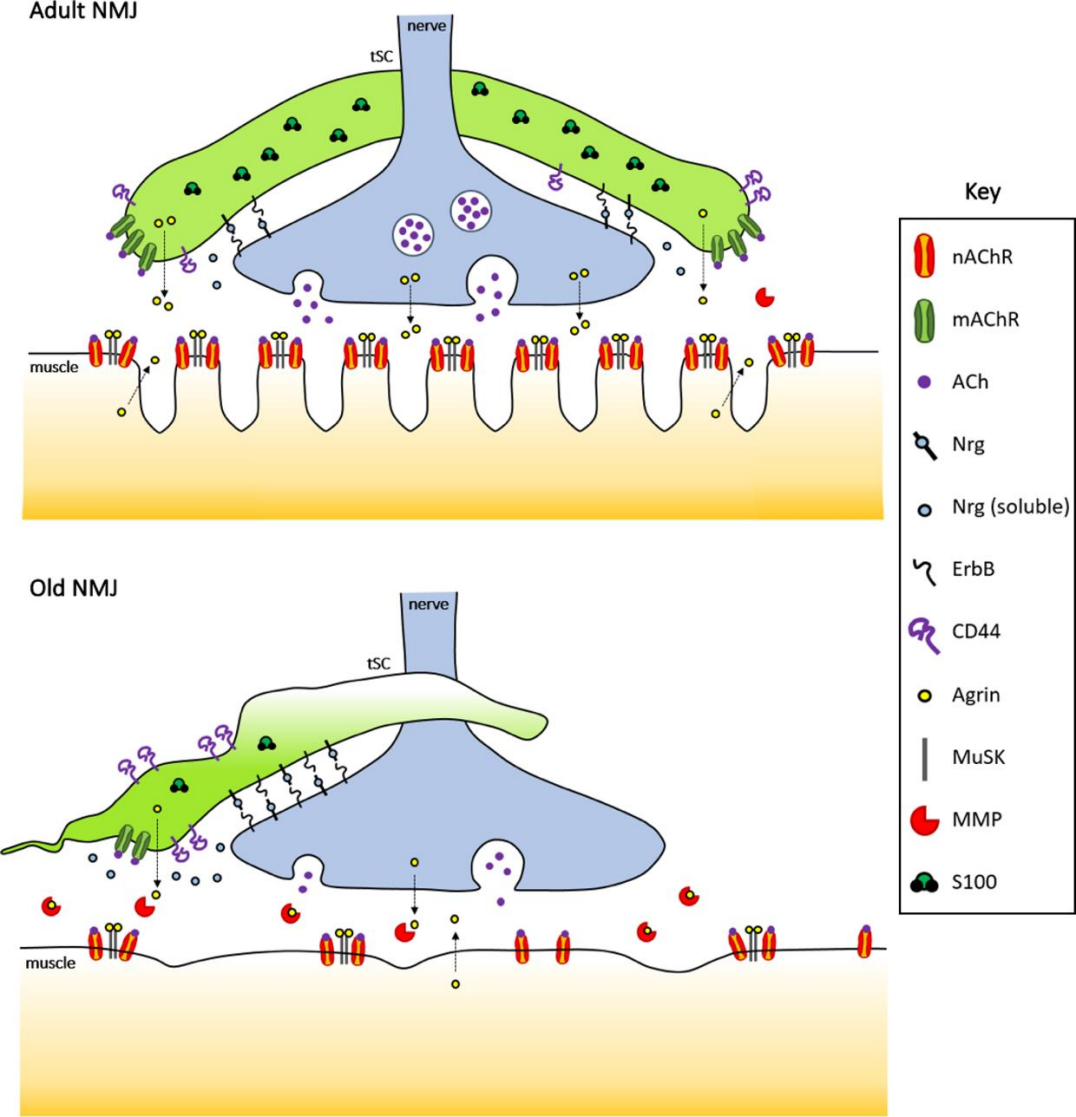
Age-associated disruption of signaling pathways at the NMJs. Normal adult (upper panel) and aged (lower panel) neuromuscular junctions are depicted showing major dysregulated pathways. NRG-ErbB -mediated signaling, implicated in NMJ stability and sprouting of tSCs, could also be involved in the migration of tSCs during aging that results in partial coverage of the NMJ. The cell surface glycoprotein CD44 seems to increase tSC plasticity, at least in ALS and aging mouse models. Agrin-MuSK pathway is essential for NMJ maintenance. The dysregulation of agrin levels in tSCs could induce NMJ destabilization during sarcopenia. Intracellular S100 proteins present altered expression in aged tSCs, which in turn affects Ca^2+^-mediated signaling.

Another controversial point, as stated above, is whether NMJ structure degenerates in elder individuals [109-111]. Classical studies described a strong age-related degeneration of human NMJs, as seen also in experimental animals [110, 111]. These studies revealed a severe fragmentation of human NMJs during aging, as well as a considerable alteration of the synaptic clefts with age. Meanwhile, a more recent study by Jones et al. maintains that human NMJs remain stable during aging [109]. One interesting point revealed by this study is the fundamental differences between NMJs in humans and mice in terms of size and shape. They analyzed lower limb muscles (extensor digitorum longus, soleus, peroneus brevis and peroneus longus) after amputation. No differences in NMJ size nor fragmentation were observed during aging when all NMJs of all muscles were clustered together. However, a separate analysis of each muscle might have been more informative to account for diverse fiber-type composition in these muscles. For instance, soleus presents mainly slow-twitch fibers [119]. In mice, fast-twitch NMJs show a more aggressive degeneration than slow-twitch NMJs [112]. Another study by Boehm et al. addressed if changes in NMJ morphology arise in chachectic patients as compared with weight stable cancer patients and controls [120]. While no significant difference was found among groups, NMJs widely varied intra- and interindividually with regard nerve terminal and endplate areas. Thus, the subject of age-associated human NMJ morphological changes deserves further scrutiny. Perhaps the categorization of NMJ degeneration variables should be adjusted for changes that seem to be specific of human NMJs and which still might possibly cause partial loss of function, such as shrinking of the NMJ surface area without apparent fragmentation. Also, issues regarding muscle tissue availability for early processing after retrieval and biopsy methods should be further refined [121].

In aged NMJs, 80% of the AChR-clusters have completely lost contact with tSCs, with only 20% of the NMJs being capped by tSCs [96]. Additionally, capping surface is reduced and NMJs are partially covered in most of the cases (Fig. 1, b) [45, 106]. Moreover, aged tSCs show a completely altered structure with characteristically thin and disorganized processes [45, 107], sometimes even with aberrant presence of tSCs sprouts in the synaptic cleft [111]. Furthermore, variable S100 expression has been described [96] (Fig. 2), possibly indicating inappropriate response to calcium-mediated signals. All these anomalies observed in aged tSCs could explain, at least in part, the high percentage of denervated NMJs in aged muscles, and provide the perfect scenario to hinder the reinnervation of denervated NMJs during aging [36, 105]. However, mechanisms involved in tSC-sprouting and their progressive degeneration during aging remain to be understood.

tSCs are extremely sensitive to the NMJ status. During nerve retraction, tSCs drastically upregulate several proteins involved in cell structure and cell junctions such as glial fibrillary acidic protein (GFAP), growth-associated protein-43 (GAP-43), low-affinity nerve growth factor (NGF) receptor p75NTR, Nestin, and cell adhesion molecule CD44 among others [22, 122]. It has been described that the number of tSC-bridges and nerve-sprouting is regulated by synaptic activity [54, 123-125]: tSCs could be detecting the synaptic microenvironment of the NMJ and determining whether to form sprouts toward adjacent NMJs, strongly suggesting the existence of a link between synaptic activity and tSC-mediated repair of the NMJ. However, the protein-expression pattern of aged-tSCs and the connection with progressive impairment of the re-innervation process during aging remains unknown.

Since tSCs are essential in maintaining the structure and function of NMJs and play key roles as sensors of synaptic communication and during NMJ-reinnervation, we believe that analyzing the signaling pathways affected in age-related degeneration is of utmost importance.

## 4.Intrinsic pathways altered in aged tSCs

tSCs play key roles in the NMJ-maintenance and function, however, both conditions are altered during aging. In this section, we discuss diverse molecular pathways which could be involved in the age-related NMJ-degeneration from a “tSchwanncentric” point of view.

### 4.1.Neuregulins (NRGs)

Neuregulins (NRGs) are a family of proteins that are involved in the development and function of the mature nervous system and the impairment of NRG-signaling contributes to neurological disorders [126-128].

NRGs function as ErbB2 receptors and are implicated in Schwann cell differentiation, proliferation, migration and myelination[127-131]. It has been described that NRGs promote tSC process extension [132, 133], which is in turn required for the formation of new neuromuscular synapses and reinnervation [99]: specifically, NRG1 is essential for tSCs collateral spouting during NMJ-reinnervation process [62, 65, 66].

NRGs are essential in NMJ-maintenance [66]: NRGs are expressed by motor neurons and tSCs at the NMJ (Fig. 2), inducing synthesis and clustering of postsynaptic nAChRs in muscle fibers [134]. Moreover, NRGs are involved in synapse elimination, plasticity of tSCs at NMJ, and migration of tSCs between endplates [132, 133, 135, 136]. Therefore, the integrity of the NMJ depends on the stability of tSCs at least in part through the regulation of NRG-ErbB signaling. In fact, bipartite NMJs lacking Schwann cells can be established only when muscle activity is blocked by ablation of downstream receptors, and the resulting NMJs showed increased spontaneous synaptic activity[137].

Similar tSC-plasticity and nerve-migration has been observed in ALS mouse models [36] and during aging [15]; thus, the NRG-pathway could be implicated in the denervation-reinnervation cycle in aged-NMJs through its regulatory role in tSCs. NRGs may be essential for maintaining clustered-AChRs during aging, and the correct expression of NRG in tSCs could be necessary for the regulation of cell sprouting and migration during denervation-reinnervation process in aged muscles.

NRG1 treatment has been proposed as a possible therapeutic candidate for peripheral nerve regeneration after nerve injury due to its role in myelinization [138], but it may also take part in re-stabilization of NMJs. Moreover, it has been described that NRG1-treatment in ALS mouse models enhances collateral tSC-sprouting to neighboring endplates [139].

Altogether, these data indicate that this family of proteins could act as potential regulators of tSCs morphological plasticity during aging.

### 4.2.CD44

CD44 is a cell surface glycoprotein which regulates cell-cell and cell-matrix interactions in several tissues [140, 141]. It is involved in cell migration [142, 143], it is able to promote cell invasion into a hyaluronan-rich matrix [144] and promotes the formation of microtentacles to facilitate migration [145].

CD44 is strongly expressed by tSCs at the NMJs (Fig. 2), and it could play an essential role in the NMJ denervation-reinnervation process via tSC-morphological plasticity regulation [146]. In ALS mouse models, tSC activation and plasticity is associated with a strong increase in CD44 expression [146], which could reflect, at least in part, what happens in tSC sprouting during aging.

Moreover, it has been proposed that NRG-ErbB signaling pathway acts through interaction with CD44 [8, 147], an interesting feature since tSCs also express ErbB receptors [146, 148]. In Schwann cells of developing peripheral nerves, CD44 constitutively associates with ErbB2 and ErbB3, where it is required for receptor heterodimerization [147]. Since both CD44 and ErbB are important regulators of morphological plasticity and cell migration [142-145, 149-152], the NRG-ErbB-CD44 axis may be involved in the regulation of tSC morphological plasticity, migration and phenotypic stability during adulthood and aging.

### 4.3.Agrin-MuSK

Agrin-MuSK (Muscle-Specific Kinase) signaling pathway is essential in NMJ formation and maintenance, and is involved in synapse elimination and in the reinnervation process of NMJs [153-159].

Agrin is expressed at the NMJs by nerve terminals, muscle fibers and tSCs (Fig. 2), where it is essential for clustering of ACh-receptors in muscle fibers [155, 160-164]. While the agrin isoform produced by terminal axons is more potent in promoting AChR clustering [161, 162, 164], the isoform produced by tSCs also seems to induce clustering [164]. To further complicate matters, muscle fibers also express agrin at the NMJs. However, muscle-derived agrin seems to be dispensable for AChR aggregation [162, 163, 165]. Experimental mouse models with impaired agrin expression show fragmented NMJs, mimicking aged NMJs and precocious sarcopenia [154]. Inactivation of PTEN and overexpression of EGFR downregulated agrin signaling and induced NMJ malformation as well as impaired autophagy [166]. On the other hand, Dok-7, an activator of MuSK, enhances neuromuscular transmission when overexpressed in transgenic mice, with concomitant increased penetration of tSC processes into the synaptic clefts [167]. Similarly, an increase in Agrin-MuSK pathway activity in ALS mouse models delays muscle denervation [168]. Therefore, these data suggest a link between agrin levels and NMJ fragmentation during aging and ALS [36, 154]: dysregulation of agrin levels in tSCs during aging could be implicated in the alteration of the pretzel-like structure, denervation and tSCs detachment observed in aged-NMJs. Moreover, the agrin partner MuSK also decreases during sarcopenia [42], further supporting a key role of agrin-MuSK pathway during aging.

Furthermore, matrix metalloproteinases (MMPs) regulate agrin levels by degradation [159]. Several studies indicate that MMP expression increases during aging [169-171], impairing motor function [172-174]. Since tSCs express MMPs [175], aged-tSCs may regulate the concentration of agrin by increasing MMP expression levels (Fig. 2), similar to what happens in NMJ development [176]. Of note, the composition and regulation of the surrounding extracellular matrix will also have a key role in the proper functioning of the NMJ [177].

In accordance with the aforementioned data, agrin-MuSK signaling axis has been proposed as a therapeutic target for myasthenia gravis and other neuromuscular disorders [178], thus reinforcing the importance of unveiling the role of agrin-MuSK pathway in tSCs during aging.

### 4.4.S100

The S100 protein family, belonging to a group of calcium-binding cytosolic proteins [179, 180], is involved in numerous biological processes such as cell cycle progression, cell differentiation, regulation of cell motility, migration, protein phosphorylation, inflammation, signal transduction and calcium balance [181-183], controlling the activity of its target proteins in a Ca^2+^-dependent manner [184]. The expression of S100 proteins has been used to characterize glial cells in the peripheral nervous system (PNS) as well as the tSC in NMJs (Fig. 2) [75, 185].

ACh in the synaptic cleft is detected by tSCs through mAChRs located in their membrane. The activation of muscarinic receptor gives rise to the activation of multiple signaling effectors which lead the regulation of intracellular Ca^2+^ via inositol-trisphosphate (IP3) [186, 187].

During aging, S100 expression is significantly reduced in old nmSCs versus young nmSCs [188] and fluctuating S100 expression has been observed in the few remaining aged-tSCs that cap NMJs in old mice [96] (Fig. 2). Dysregulation of S100 expression in tSCs could give rise to anomalies in the detection of intracellular Ca^2+^. Thus, aged-tSCs could somehow be “misunderstanding” the synaptic signal and modulating it in a dysfunctional manner.

### 4.5.TGF-β

TGF-β superfamily proteins are one of the mayor extracellular regulators, and they are widely expressed in the nervous system [189]. The TGF-β pathway plays key roles in myelinating-SCs biology [190-192] and during regeneration after nerve injury [193] but also in tSCs: it has been observed that Schwann cell-conditioned medium induces synaptogenesis in NMJs in vitro via TGF-β [194]. It has also been suggested that tSCs release TGF-β, which in turn affects NMJ assembly and maturation in vivo [192].

TGF-β is also a local regulator of NMJ activity [195], and suppresses the expression of the protein FGFBP1 (Fibroblast Growth Factor-Binding Protein 1) [196], which is implicated in the maintaining of NMJ structure: increasing levels of TGF-β in the NMJ decrease FGFBP1 expression, which correlates with a degeneration of the NMJ structure [196]. Therefore, proper regulation of TGF-β expression levels seems to be essential for NMJ stability, and the alteration of TGF-β expression could be implicated in the NMJ disaggregation during ALS and aging. Therefore, it is essential to determine the TGF-β expression in aged-tSCs in order to unveil, at least in part, the signaling pathways involved in NMJ-degeneration during aging.

### 4.6.Growth-Associated Protein-43 (GAP-43)

GAP-43 is a membrane-associated phosphoprotein involved in the regulation of neurite outgrowth in developing and regenerating neurons both in the PNS [197-199] and the CNS [200]. GAP-43 is also expressed by skeletal muscle fibers, where it is probably implicated in the regulation of Ca^2+^-homeostasis [201, 202]. More relevant for the purpose of this review, it is also expressed in both myelinating-SCs [203, 204] and non-myelinating SCs [199, 204, 205] after nerve injury. Specifically, GAP-43 is expressed in tSCs at the NMJ after nerve injury-mediated denervation, and its expression depends on the neural contact [76]. Functional denervation after botulinum toxin treatment increases GAP-43 expression in motor neurons but not in tSCs at the NMJs [198]. Thus, GAP-43 plays a key role in the detection of tSC-terminal nerve contact and could be acting as a sensor in the case this connection is deficient.

During aging, a reduction in GAP-43 levels in the CNS is observed [200, 206], which suggests an age-associated loss of synaptic plasticity. However, and in spite of its essential role in the regulation of neurite outgrowth, the patterns of GAP-43 expression in the PNS and in NMJs during aging remain to be understood.

## 5.Extrinsic pathways affecting aged tSCs

Aging affect numerous pathways at the organismal level. The NMJ is obviously exposed to environmental inputs that will extrinsically induce reorganization of its structure and function, and what is known on extrinsic pathways will be reviewed in this section.

### 5.1.Effect of sirtuin Sirt1 expression on tSCs structure

Sirt1 is a protein involved in the control of biological processes such as cell survival, senescence and proliferation during aging [207-210]. It has been observed that hypothalamic Sirt1 regulates aging and longevity. Overexpression of Sirt1 extends the lifespan of mice, and specifically delays aging of skeletal muscles, maintaining a youthful appearance [211-213].

Interestingly, a recent study suggests that hypothalamic Sirt1 could be involved in the protection of the NMJs from age-related changes. Specifically, overexpression of Sirt1 in the brain of aged mice (BRASTO mice) [214, 215] correlates with a higher percentage of innervated NMJs, less fragmented AChR clusters and increased number of NMJs covered by tSCs. These data suggest that the sirtuin pathway could be indirectly modulating age-related NMJ degeneration through a possible protective effect on aged tSCs [96]. However, at present the evidence is correlative and causal studies are lacking.

### 5.2.Effect of inflammaging on tSCs

Inflammaging is defined as the chronic low-grade inflammation observed in diverse organs and tissues in elderly people. It is characterized by the sustained expression of inflammatory mediator proteins, such as interleukin 6 (IL-6), interleukin 1 (IL-1), tumor necrosis factor alpha (TNF-α) and C-reactive protein (CRP) [216-221]; as well as by macrophage infiltration [222]. In skeletal muscle, inflammaging is associated to muscle wasting and weakness, thus accelerating a decline in muscle mass and strength, and promoting age-associated mobility disability [223-227].

How does this pro-inflammatory state affect NMJ functionality? Schwann cell senescence and limited axonal regeneration result from overexpression of IL-6 [228]. Thus, tSCs might become senescent due to inflammaging [229]. Moreover, it has been described that aged mice present poor peripheral nerve regeneration after injury compared with young animals [229-231]. However, intrinsic nerve growth capacity is not affected by aging [232]. Considering that SCs are essential in guiding nerve sprouts after nerve damage [233, 234], SC senescence could underlie, at least in part, nerve regeneration impairment in inflammaged muscles. Moreover, inflammaging could induce tSC death, thus irreversibly affecting NMJ maintenance and the formation of “bridges” to adjacent endplates. Aging also impairs the function of macrophages and other immune cells [222, 235-237]. Both macrophages and SCs are essential in clearing debris during nerve regeneration upon injury [233, 238, 239]. As a result, nerve regeneration in aged muscles may suffer from inefficient clearance of debris [229-231]. Whether the presence of macrophages or their impaired functionality affect tSC bridge formation in aged muscles remains unknown.

As aforementioned, another molecule involved in inflammaging is TNF-α. Interestingly, overexpression of TNF-α in postsynaptic muscle cells depurates supernumerary NMJs by inducing the retraction of redundant nerve terminals [240]. One can posit that TNF-α may also induce nerve terminal retraction, giving rise to partially innervated NMJs and denervated NMJs as a consequence of inflammaging. In any case, inflammaging of the local environment of the NMJ possibly induces degeneration through several of the above mechanisms in a concerted fashion. However, the present evidence is scarce and further research is needed to move from speculative ideas to certainties.

## 6.tSC implications on the mechanisms of muscle aging and sarcopenia

In aged individuals, muscle age-related degeneration implies the gradual loss of muscle strength (dynapenia) and muscle mass (sarcopenia), which together lead to loss of muscle function [42, 108, 117, 241-246]. The strength reduction occurs prior to significant muscle mass loss [247]. These age-associated alterations impair physical ability in older adults and are associated with progressive skeletal muscle atrophy, denervation and loss of muscle fibers (especially type II fibers or fast fibers), motor neurons loss and accumulation of fat within muscle [13, 42, 248, 249]. The age-derived degenerative processes give rise to weakness, mobility limitations, frailty and a high risk for falls [115, 250, 251], strongly affecting the quality of life of elderly people.

Skeletal muscle fibers undergo repeated cycles of denervation-reinnervation during adult life [252] : once muscle fibers undergo denervation, they express chemotactic signals that induce reinnervation by the extension of proximal motor nerve terminals [253, 254]. These denervation-reinnervation cycles, repeated throughout adult life, lead to remodeling of the motor units [42, 255-257], disrupt the precise overlapping between the pre-synaptic nerve terminal and the post-synaptic receptors (AChRs) at the NMJs [258], and give rise to alterations in nerve terminals and in the distribution of laminin [45, 108, 157, 259]. The dynamics of denervation-reinnervation cycles begins to fail with age [257] because denervation outpaces reinnervation. As a consequence, subsets of denervated fibers are not successfully reinnervated [42, 257, 260]. Preferential denervation of the fast-twitch (type II) fibers takes place in animal models [261-264]. Loss of motoneurons has also been observed in humans [265-267]. Denervated fibers that are not successfully reinnervated undergo atrophy [42, 249, 268], thus contributing to mobility impairment and physical frailty.

Some fibers are reinnervated by axonal sprouting from slow motor neurons (that innervate slow-twitch fibers or type I fibers), which lead to remodeling of the motor units and resulting in fiber-type grouping and in a preponderance of type I motor units in aged muscles [42, 255, 256, 269], contributing to the loss of muscle strength since fast-twitch motor units determine the degree of power exerted by the underlying muscles. All these degenerative processes ultimately compromise the contractile function of the skeletal muscles during aging. The preferential denervation of type II fibers explain, at least in part, why people with a sedentary lifestyle are more susceptible to sarcopenia than people with an active lifestyle [42, 270, 271], since fast-twitch fibers are responsible for the power exerted by muscles. The disuse of type II motor units due to a limited use of explosive actions would accelerate their denervation, atrophy and degeneration.

Recently, age-associated changes of NMJs have been strongly implicated in the loss of muscle mass and strength during aging, since NMJ age-associated remodeling and denervation precedes muscle atrophy [12, 13, 46, 272]. On the contrary, Slater has proposed an alternative view, in which NMJ fragmentation would be the outcome of a functional regenerative process [273]. His argument is based on the lack of evidence for age-related impairment in neuromuscular transmission, despite the NMJ undergoing morphological changes [273, 274]. To clarify matters, it would be essential to determine the origins of this controversy. Methodological aspects that differ among studies, such as different types of muscle fibers [112, 275-279], diverse localization (proximal or distal muscles) [280], or different lifelong usage of the skeletal muscles analyzed may underlie some of the observed differences. Also, how “aged” animals are defined will have an impact: a recent study found that NMJ transmission defects arose as late as 27-29 months in C57BL/6 mice [281]. The susceptibility of NMJs to suffering from denervation and the ability to be reinnervated depends largely on the morphological plasticity of tSCs. As discussed above, tSCs are essentially involved in maintaining the structure and function of NMJs in homeostatic conditions, but they also play an important role in axonal guidance and synaptic repair after denervation, even during aging [99, 105, 106]. Moreover, the impairment of tSCs gives rise to NMJ-fragmentation and denervation [30]. Therefore, it is plausible that aged tSCs may contribute to age-related NMJ denervation and inefficient re-innervation [13]. There have been scarce reports about age-associated tSC degeneration [45, 96, 107]: aged tSCs show disorganized appearance, altered structure that covers partially the endplate, and even absence in large part of NMJs. However, the implication of tSC aging during denervation-reinnervation cycles remains to be understood.

In addition, altered Wnt pathway might also be involved in age-associated NMJ disruption, as seen in muscle development. During development, Wnt signaling regulates NMJ formation, as it is essential for AChR clustering [14, 282-290]. Wnt ligands and Wnt-related proteins are expressed by muscle cells, nerve terminals and tSCs [260, 289-295], with possible redundant and compensatory functions at the NMJ local environment. Downregulation of Wnt upon skeletal muscle aging may signal a progressive reduction in muscle regenerative capacity [296, 297]. However, activation of Wnt in aged myogenic progenitors is also detrimental since it directs them to a fibrogenic lineage [298]. A correlation between Wnt pathway activation and fibrosis has also been observed in other aged tissues [299]. Although the regulation of Wnt signaling in aged tSCs is unknown, it seems like a worthy area of investigation.

We propose tSCs are not only a crucial element in adult NMJ maintenance and function, but also an essential player in the aged neuromuscular synapse, where age-related degeneration of tSCs could be responsible for the denervation of muscle fibers. It is thus essential to analyze tSCs as clinical targets to avoid NMJ-denervation and/or enhance the NMJ-mediated muscle reinnervation during aging.

## 7.Concluding remarks

The increase of the life expectancy of the population in the developed world is leading to a dramatic growth of the prevalence of age-related muscle degeneration. Therefore, prevention and treatment of sarcopenia and dynapenia have emerged as fields of high medical need in order to improve the quality of life of the elderly people. Studying NMJ-associated changes during aging would let us better understand the age-related degeneration of skeletal muscles and sarcopenia. Specifically, deciphering the tSC behavior during aging would shed light on the mechanism of muscle denervation.

Morphological and physiological alterations of tSCs during aging could be implicated in the denervation process and even in the muscle remodeling observed during aging, where a preferential denervation and degeneration of type II muscle fibers takes place. The key roles of tSCs in NMJs during sarcopenia define them as perfect therapeutic targets to avoid or delay denervation derived from aging. Therapies focused on maintaining the tSCs that cap NMJs, avoiding denervation, or even improving the formation of bridges toward denervated NMJs would be of potential medical relevance.

A potentially interesting therapeutic approach is Schwann cell therapy. Schwann cell transplantation enhances myelination and spinal nerve regeneration in the spinal cord [300-304]. Schwann cell grafts could also be a potential therapy for peripheral nerve injury [305]: SC transplantation successfully enhances sciatic nerve regeneration not only in rodents [302, 306-310], but also in monkeys [311] and in humans [312]. However, SC therapy presents the obstacle of limited cell sources. Therefore, it would be interesting to explore other possibilities such as the culture of SC precursors or human induced pluripotent stem cells (iPSCs) [313-315], in order to generate healthy stem cell-derived tSCs that keep capping NMJs during aging, thus avoiding age-related denervation. Obviously, large muscle groups present an enormous logistical challenge to the application of cell-based approaches, since it is necessary to perform multiple microinjections along the muscle to facilitate transplanted cells to reach the NMJs. However, this idea might make sense on smaller muscle groups, such as those covering both urinary and anal sphincters as well as ocular muscles. Cell therapy is already being used to treat urinary incontinence [316], oculopharyngeal dystrophy [317] and other ocular diseases [318-320], among others. Treatment of larger muscles would possibly require cell transdifferentiation techniques mediated by small molecules or viral vectors, which are simpler to deliver [321-323]. For instance, Schwann cell precursors have been generated in vitro from fibroblasts treated with episomal vectors [324]. One could envision this type of approach promoted locally in vivo, making use of fibrotic areas abundant in aged muscle. Another interesting approach is the identification of pharmacological treatments which could avoid premature denervation or improve the reinnervation process of NMJs during aging. An interesting therapeutic candidate is Fingolimod (also known as FTY720P), a synthetic drug used in autoimmune diseases: it is approved for treatment of patients with multiple sclerosis, where it is implicated in the demyelination-remyelination process in the CNS [325, 326]. In the PNS, Fingolimod seems to regulate myelin production and differentiation of Schwann cells [327]. Moreover, it is implicated in the reduction of neuro-inflammation [328] and promotes neurite outgrowth [329]. Therefore, it would be interesting to analyze the effect of Fingolimod treatment in peripheral nerve injuries and PNS-associated diseases. Once its effect on tSCs is determined for these diseases, its viability could also be evaluated for use in age-related denervation treatment. Recent studies highlight the complexity of age-related alterations of NMJ and the implications in sarcopenia, however the role of tSCs in this process has been largely overlooked. The idea that Schwann cell dysfunction may constitute a primary trigger of sarcopenia was originally proposed by Kwan a few years ago [330]. With this review, we hope to reignite the interest in this long-standing issue and help frame potentially interesting signaling pathways to explore. Identifying the molecular basis of tSCs dysfunction during aging is essential to understand the process of NMJ denervation and the basis of failed NMJ reinnervation. Developing new therapeutic strategies to counteract age-derived denervation might improve the quality of life of elderly people as well as improve our knowledge about healthy aging.
